# Regional differences in dosage compensation on the chicken Z chromosome

**DOI:** 10.1186/gb-2007-8-9-r202

**Published:** 2007-09-27

**Authors:** Esther Melamed, Arthur P Arnold

**Affiliations:** 1 Department of Physiological Science, and Laboratory of Neuroendocrinology of the Brain Research Institute, University of California, Los Angeles, CA 90095-1606, USA

## Abstract

**Background:**

Most Z chromosome genes in birds are expressed at a higher level in ZZ males than in ZW females, and thus are relatively ineffectively dosage compensated. Some Z genes are compensated, however, by an unknown mechanism. Previous studies identified a non-coding RNA in the male hypermethylated (MHM) region, associated with sex-specific histone acetylation, which has been proposed to be involved in dosage compensation.

**Results:**

Using microarray mRNA expression analysis, we find that dosage compensated and non-compensated genes occur across the Z chromosome, but a cluster of compensated genes are found in the MHM region of chicken chromosome Zp, whereas Zq is enriched in non-compensated genes. The degree of dosage compensation among Z genes is predicted better by the level of expression of Z genes in males than in females, probably because of better compensation of genes with lower levels of expression. Compensated genes have different functional properties than non-compensated genes, suggesting that dosage compensation has evolved gene-by-gene according to selective pressures on each gene. The group of genes comprising the MHM region also resides on a primitive mammalian (platypus) sex chromosome and, thus, may represent an ancestral precursor to avian ZZ/ZW and monotreme XX/XY sex chromosome systems.

**Conclusion:**

The aggregation of dosage compensated genes near the MHM locus may reflect a local sex- and chromosome-specific mechanism of dosage compensation, perhaps mediated by the MHM non-coding RNA.

## Abstract 2

Microarray data analysis revealed a cluster of well compensated genes in the MHM (male-hypermethylated) region on chicken chromosome Zp, whereas Zq is enriched in non-compensated genes.  The non-coding MHM RNA may therefore play a role in dosage compensation in the female.

## Background

In birds, males are homogametic (ZZ) and females are heterogametic (ZW), in contrast to the mammalian pattern of female XX homogamety and male XY heterogamety. Like the mammalian X and Y chromosomes, the euchromatic Z is large (over 500 genes) and the heterochromatic W small (probably containing tens of genes) [1-4]. In both groups, the difference in copy number of the Z or X chromosomes results in one sex having a higher genomic dose of Z or X genes.

Gene dosage is considered to be critical, at least for a significant number of genes, and an imbalance in chromosomal number (aneuploidy) can result in conditions such as Turner syndrome (XO), Klinefelter syndrome (XXY), Down syndrome (Trisomy 21), and cancer [5-7]. Aneuploidy for an entire chromosome is usually lethal [8]. Because a delicate balance in gene dosage is important for proper functioning and organismal survival, numerous species have evolved mechanisms of sex chromosome dosage compensation, which balances the expressed dose of X genes between males and females, and between the X chromosome and the autosomes. Mammalian dosage compensation is accomplished through inactivation of one of the X chromosomes in every female cell, and upregulation of the single active X chromosome in both males and females [9,10]. These combined mechanisms effectively equate the expressed gene dose between the X chromosome and autosomes [9,11]. Other XX-XY species have evolved different effective dosage compensation mechanisms. For example, *Drosophila *XY males upregulate gene expression from their single X chromosome to bring the dose on par with that of the XX female and with autosomes [12,13]. In *Caenorhabditis elegans*, XO males and XX hermaphrodites both upregulate X gene expression and the hermaphrodite downregulates each X chromosome, again resulting in compensation of X genes between the sexes and with autosomes [8,12].

In birds, however, Z genes are not as well compensated as are X genes in mammals, flies, and worms [11,14-19]. Z mRNAs are expressed about 30-40% higher in chicken ZZ males than in ZW females [11]. In addition, several Z genes appear to be expressed biallelically, suggesting that inactivation of an entire Z chromosome does not occur in males [20,21]. Nevertheless, an unknown type of dosage compensation mechanism results in sexual parity of expression for some Z chromosome genes, bringing the Z to autosomal ratio of expression to around 0.8 in ZW female chickens [11,15].

The mechanism of dosage compensation on the Z chromosome is unclear. In chickens a female specific non-coding RNA is expressed from the male hypermethylated (MHM) locus and accumulates near its transcription site on the Z chromosome [22]. In ZZ males, the DNA at the MHM locus remains hypermethylated and untranscribed [22]. In addition, histone H4 at lysine residue 16 is acetylated in a region surrounding the MHM locus in ZW females but not in ZZ males [23]. Other non-coding RNAs, such as XIST in mammals and roX genes in *Drosophila*, are implicated in the control of X chromosome dosage compensation. Moreover, histone H4 acetylation is associated with dosage compensation in *Drosophila *[24]. These observations suggest that the MHM RNA and female-specific histone acetylation may lead to hypertranscription of Z genes in females, which could compensate Z dosage [23]. It is not clear, however, whether a dosage compensation mechanism occurs in only one or in both sexes, and which parts of the Z chromosome might be affected. The involvement of MHM and its associated histone acetylation in dosage compensation has, heretofore, received no direct support.

By mapping the male to female ratios of mRNA expression of Z genes according to their positions on the Z chromosome, we report here that dosage compensated genes are located all along the chromosome, but that the MHM region contains a higher percentage of compensated genes than other regions. Compensated genes show signs of having significantly different functional properties than genes that are not compensated, suggesting that dosage compensation has evolved according to selective pressures on individual genes. We propose that detrimental effects of a lack of overall dosage compensation on the Z chromosome may be mitigated by selective compensation of genes that are most dosage-critical, both in the MHM region and elsewhere on the Z chromosome.

## Results

### Regional variation of dosage compensation

**Figure 1:**
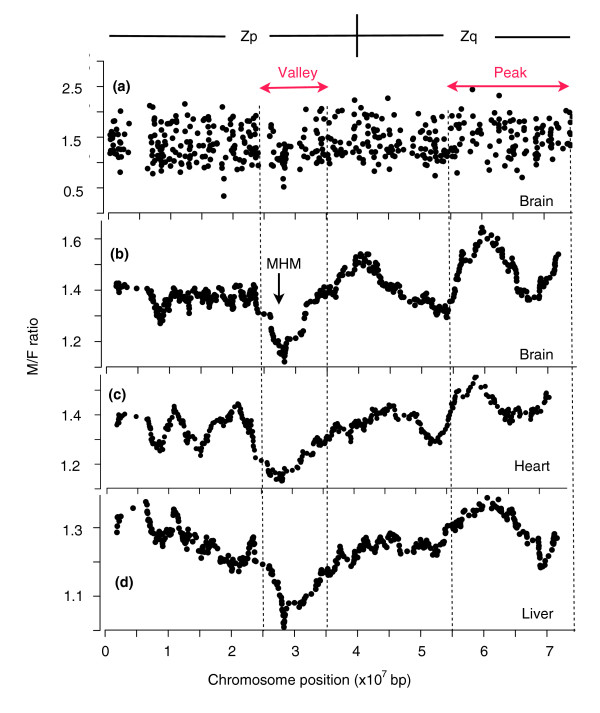
M:F ratios as a function of Z chromosome position for brain, heart, and liver tissues. **(a) **Individual M:F ratios in the brain, graphed by gene position on the Z chromosome. **(b-d) **The running average of 30 M:F ratios is plotted at the median gene position, for brain, heart, and liver. The curves all show a dip, or valley, surrounding the MHM locus of Zp, comprising a cluster of dosage compensated genes in a region deficient in genes with high M:F ratios, as well as an elevated region (one or two peaks) at the distal end of Zq with an unusual concentration of non-compensated genes.

We measured mRNA expression in brain, heart, and liver of male and female chick embryos at 14 days of incubation, based on microarray analysis (see [11]). In each tissue, the male:female (M:F) ratio of mRNA expression was calculated for each gene. We have previously reported that Z genes are expressed at higher M:F ratios than autosomal genes, and that some Z genes appear to be dosage compensated (M:F ratios in the range 0.8-1.3, for example) whereas many others are not (for example, ratios above 1.5) [11]. To determine whether genes showing dosage compensation are concentrated in specific regions of the Z chromosome, we mapped M:F ratio by gene position along the Z chromosome (Figure 1a). The map indicates that genes with high and low M:F ratios are found across the entire Z chromosome. To accentuate trends in the amount of dosage compensation, we computed a running average of M:F ratios as a function of position on the Z chromosome (Figure 1b). We observed two major features in the running average curve: a dip ('valley') and a broad peak corresponding to areas rich in compensated and non-compensated genes, respectively. These features were independent of the number of genes averaged to smooth the curve. The valley was not produced by a small number of genes with exceptionally low M:F ratios, but rather was formed because of the relative lack of genes with high M:F ratios in all three tissues (for example, Figure 1a). The peak resulted from an over-representation of high M:F ratios. The valley was located on Zp near the centromere and overlapped with the MHM locus (NCBI CoreNucleotide accession number AB046699), whereas the peak was found on the distal end of Zq. The graphs for three tissues showed highly similar peaks and valleys (Figure 1b-d), suggesting that regulation of M:F ratios occurs globally, perhaps by a regional mechanism on the Z chromosome, and not just by tissue-specific factors. The approximate boundaries of the Zp MHM valley (2.5E7 to 3.5E7 bp) and Zq peak (5.5E7 to 7.5E7 bp) were estimated visually based on common inflection points in the three curves (Figure 1b-d). In the datasets of genes expressed in brain, heart, or liver, valley genes accounted for 61 and peak genes for 121 of 504 expressed Z genes (Additional data file 1). The mean M:F ratio for Zp genes was significantly lower than the mean M:F ratio for Zq genes in the three tissues (*p *< 0.004 for brain, *p *< 0.00083 for heart, and *p *< 0.045 for liver, *t*-test).

To test whether the peaks and valleys of the three curves occur by chance, for each tissue we counted the number of consecutive positions on the graphs in Figure 1b-d that remain below the 40th percentile of Z M:F ratios in the Zp valley, or remain above the 60th percentile of Z M:F ratios in the Zq peak. Then we shuffled the M:F ratios of genes randomly 10,000 times for each tissue and found that consecutive runs of low valley values and high peak values occurred rarely together on each shuffled chromosome in the permutation analysis (*p *< 0.0001 in each tissue). The valley occurred rarely (*p *≤ 0.001) by chance within 30 positions of the MHM locus (*p *< 0.004) or indeed anywhere on the chromosome (*p *≤ 0.001) in each tissue.

**Figure 2:**
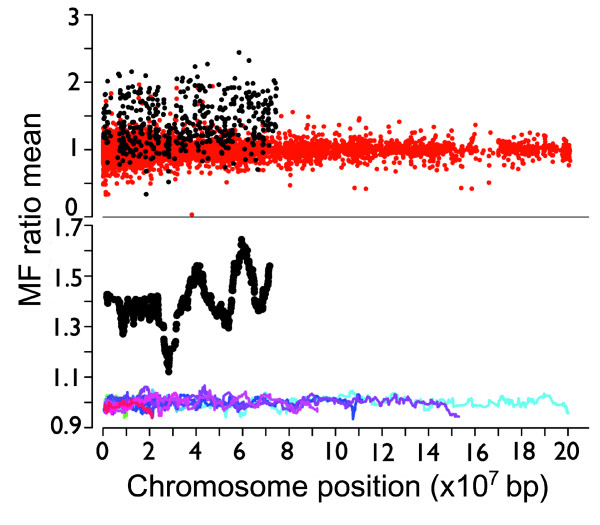
Running average of M:F ratios on the Z chromosome and autosomes. Top: plot of the M:F ratio of individual Z genes in brain (black) compared with the expression genes for 18 autosomes (red) containing more than 200 genes. Bottom: the running average of M:F ratios (calculated as in Figure 1) for brain shows that the Z chromosome (black) has much more pronounced valleys and peaks than are found in the autosomes (red through blue).

To determine whether the valley and peaks were unique to the Z chromosome, we calculated the running averages of M:F ratios for 18 autosomes with at least 200 expressed genes each. In contrast with the Z chromosome, autosomal gene M:F ratios were similar to each other, with means near 1, but with no peaks or valleys comparable to those on the Z chromosome (Figure 2).

Non-linear measurement of expression by microarrays could theoretically lead to underestimates of M:F ratios in regions of the chromosome that contain genes with low or high levels of expression. We previously used quantitative PCR to confirm M:F ratio accuracy in the microarray dataset [11]. To test whether the unusual cluster of dosage compensated genes in the MHM valley could result from low gene expression levels, we compared gene expression levels across the Z chromosome and on chromosome 1 (Additional data file 6). We found that other regions of the Z chromosome have equal or lower average expression levels, and that the MHM valley and Zq peak are not well predicted by the level of expression of genes. Moreover, Z expression levels were comparable to those on autosomes, where M:F ratios are near 1. Thus, the regional variation in M:F ratios appear not to be an artifact of regional differences in expression levels.

### Correlation of compensation with sex-specific gene expression

**Figure 3:**
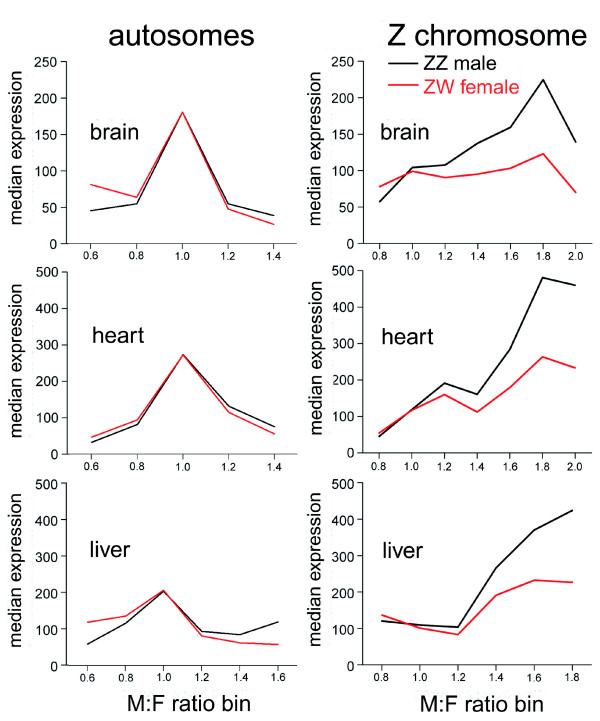
ZZ male and ZW female expression levels as a function of M:F ratio. Graphs show the median expression level of genes as a function of M:F ratio. For each tissue, all genes showing expression from the Z chromosome or from autosomes 1-5 were grouped into bins according to M:F ratio. The graphs indicate that autosomal genes with high (>1.2) or low (<0.8) M:F ratios show generally lower expression, in both sexes, relative to genes that are equally expressed in the two sexes (M:F ratio near 1), indicating that sexually dimorphic expression is not associated with higher expression in one sex relative to the majority of genes. Among Z genes, expression in females varies little as M:F ratio changes. Male genes, however, are expressed significantly lower at low M:F ratios near 1, relative to higher M:F ratios. Bin width is 0.2. Values are plotted at the mid-point of the bin. A small number of genes, with M:F ratios outside of the range shown, are included in the most extreme bins.

**Figure 4:**
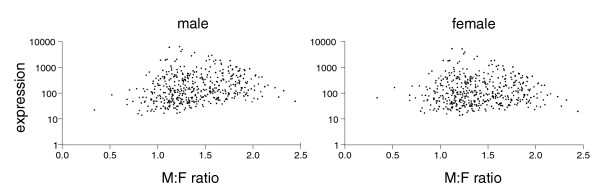
Relationship between male and female gene expression and M:F ratio in brain. The level of expression of each gene in brain is plotted as a function of M:F ratio for each sex, to illustrate the correlation of the two variables in males but not females.

We examined variation in expression levels of Z genes in the two sexes for evidence of gene regulation that might reflect a sex-specific mechanism of dosage compensation. In ZW females, the average level of expression of dosage compensated and non-compensated genes was about equal to the average for the entire Z chromosome, but in ZZ males compensated genes were expressed on average at a lower level, and non-compensated genes at a higher level, than the Z chromosome average (Table 1, Figures 3 and 4). Bootstrap resampling analysis indicated that the female expression values for compensated and non-compensated genes did not differ significantly from all Z genes (*p *> 0.05), whereas the actual mean male expression values for compensated and non-compensated genes in each tissue were unexpected if the genes were drawn at random from the set of all Z genes (*p *< 0.02 in each case).

**Table 1:** Expression levels in males and females of all Z genes and compensated and non-compensated Z genes

	All Z genes	Compensated Z genes	Non-compensated Z genes
Male brain	228 ± 12	179 ± 15	295 ± 23
Female brain	162 ± 8	161 ± 13	169 ± 14
			
Male liver	366 ± 30	263 ± 32	596 ± 78
Female liver	287 ± 24	248 ± 30	363 ± 48
			
Male heart	388 ± 24	317 ± 33	567 ± 49
Female heart	280 ± 18	283 ± 28	332 ± 29

Data are mean ± standard error of the mean. Compensated genes are those with an M:F ratio below 1.3; not compensated genes are those with an M:F ratio above 1.5.

The level of expression of Z genes in each tissue in males was significantly positively correlated with the M:F ratio of Z genes (Pearson correlation coefficient r = 0.192 to 0.234, *p *< 0.0011) whereas the female expression values were not significantly correlated with M:F ratios (r = 0.013 to 0.075, *p *> 0.1) (Figure 4). *A priori*, it is not clear whether the positive correlation in the male is the result of the dosage compensation or results from another mechanism. To determine if these correlation patterns might suggest which sex possesses a compensation mechanism to adjust the dose of some Z genes, we modeled the effect of male-specific or female-specific compensation mechanisms on the correlation of gene expression with M:F ratio. In model I, the probability of dosage compensation was assumed to be unrelated to the level of expression of genes. Taking the brain gene expression values of chromosome 1 genes as a representative set of chicken genes, we assigned to each chromosome 1 gene an M:F ratio drawn at random from the distribution of Z chromosome M:F ratios. To mimic a female-specific dosage compensation mechanism, a Z M:F ratio was randomly assigned to each chromosome 1 male gene expression value. The assigned M:F ratio and the male expression value was used to calculate the corresponding female expression value for the gene. In this analysis, the female gene expression values reflected the level of compensation normally found on the Z chromosome. To model a male-specific compensation mechanism, a Z M:F ratio was assigned randomly to each chromosome 1 female gene and used to calculate the corresponding male expression level. In each case, the assignment of M:F ratios to genes of various expression levels was repeated 100 times, and in each iteration the correlation coefficient r was calculated between male expression values and M:F ratios, and female expression values and M:F ratios.

**Figure 5:**
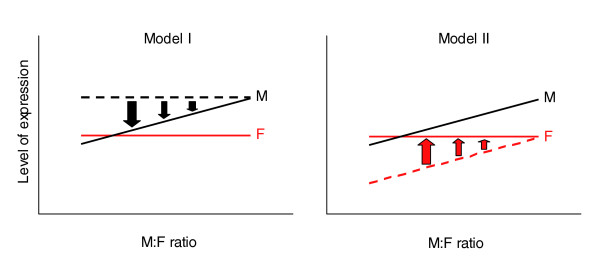
Models of sex-specific mechanisms of dosage compensation. Model 1 assumes that prior to compensation, female (red line) and male (dotted black line) expression is unrelated to the eventual amount of dosage compensation. If the male reduces expression of genes to compensate, the line tilts down on the left, resulting in a pattern of positive correlation of level of expression and M:F ratio (black solid line), close to that observed. Model II assumes that prior to dosage compensation, female (red dotted line) and male (black solid line) expression is correlated with the eventual level of compensation (lower average expression in genes to be compensated). In model II, female-specific compensation, illustrated by the shift to the red solid line in the female, leads to the observed pattern in which female expression does not correlate with M:F ratio. Arrows indicate shifts required in each model to achieve the observed pattern that male gene expression is correlated with M:F ratio, but female level of expression is not.

When a female compensation mechanism was modeled, M:F ratios were weakly negatively correlated with female expression (mean r = -0.21) and not correlated with male expression (mean r = 0.00). When a male compensation mechanism was modeled, M:F ratios were weakly positively correlated with male expression (mean r = 0.20) and uncorrelated with female expression (mean r = 0.00). Therefore, model 1 best matched the observed correlation pattern between M:F ratios and expression values on the Z chromosome when the male but not female possessed a sex-specific mechanism of dosage compensation. A male-specific mechanism means that the male down-regulates expression of some genes to match the level of expression in females (Figure 5). This mechanism, by itself, could lower average Z gene expression levels below that of autosomal genes, which conflicts with the observed Z:A expression ratio of about 1 in males [11]. Thus, the assumptions leading to model I are questionable.

In model II, the amount of compensation was assumed to increase as a function of gene expression level in both sexes (since the level of expression of individual genes in the two sexes is highly correlated across a wide range of gene expression values; Figure 4). The procedures for model II are equivalent to those for model I except that M:F ratios were assigned randomly to genes but then multiplied by an adjustment factor that incremented the M:F ratio in proportion to gene expression level. The adjustment factor set M:F ratios about 20% higher for genes at the highest expression levels relative to those at the lowest expression levels, with graded adjustment in between. When a female compensation mechanism was modeled, the M:F ratio correlated with male expression level (mean r = 0.22) but not with female expression level (mean r = -0.02). When a male compensation mechanism was modeled, M:F ratio was correlated with both male (mean r = 0.41) and female (mean r = 0.22) expression level. Thus, under this set of assumptions, a female compensation mechanism led to a correlation pattern most similar to the observed pattern.

### Functional differences among compensated versus non-compensated genes

We used two different algorithms to explore the functional characteristics of genes in different regions of the Z chromosome: Database for Annotation, Visualization and Integrated Discovery (DAVID) and Ingenuity Pathway Analysis (IPA). We first asked whether genes in the valley and peaks on the Z chromosome were enriched in specific functions (Additional data files 2 and 3). Valley genes were found to be enriched in genes involved in regulation of cellular physiological process, regulation of DNA dependent transcription, reproductive system development and function, embryonic development, developmental disorder, and cellular growth and proliferation. Peak genes were enriched for involvement in the nucleus, DNA repair and response to DNA damage, catalytic activity, and intracellular membrane bound organelle (Additional data files 2 and 3).

We also asked whether compensated and non-compensated genes, irrespective of their position on the Z chromosome, differed in gene functions (Additional data files 4 and 5). Compensated and non-compensated genes were arbitrarily defined as genes with M:F ratios less than 1.3 or higher than 1.5, respectively. Compensated genes were enriched in all three tissues for cell signaling and interaction, developmental disorder, organismal development/survival, cellular growth and proliferation, signal transducer activity in heart and brain, and receptor activity in brain. Non-compensated genes were enriched for intracellular membrane bound organelle in all three tissues, and for intracellular, chromosome, cell organization and biogenesis, and nucleus in brain (Additional data files 4 and 5).

## Discussion

Here we report that dosage compensated genes are interspersed with non-compensated genes across the entire Z chromosome of chickens. Nevertheless, a higher concentration of fully compensated genes occurs in a region adjacent to the MHM locus, which shows female-specific expression of the non-coding MHM RNA [22]. The same region is enriched in female-specific hyperacetylation of histone H4, which is postulated to play a role in balancing gene expression between the sexes [23]. The clear regional correlation of dosage compensation with female-specific RNA expression and chromatin modifications suggests strongly that the MHM non-coding RNA and/or associated histone modifications are involved in regulating dosage compensation of Z genes. The pattern suggests that compensation in the MHM valley is regulated by a chromosome-specific mechanism, as is found for sex chromosomes of other species, rather than by a gene-specific or tissue-specific molecular mechanism. The aggregation of dosage compensated genes in the MHM valley may be biologically meaningful because it is unlikely to have occurred by chance. In contrast to mammals, *Drosophila*, and *C. elegans*, in which sex chromosome dosage compensation is chromosome-wide, chicken dosage compensation appears to occur in a greater percentage of genes in a relatively small region of Zp, suggesting that the DNA in this region is specialized. It is intriguing that a general sex-specific and multi-genic molecular mechanism of dosage compensation might exist in birds, but not have spread to all regions of the sex chromosome as occurs in various XX-XY systems. Of course, if the MHM RNA is centrally involved in Z dosage compensation, its effects may also occur beyond the MHM valley, and contribute to compensation of specific genes in other Z regions. The concentration of dosage compensation in the MHM valley, compared with chromosome-wide mechanisms in other species, raises the question of what evolutionary forces account for these differences in dosage compensation mechanisms in various taxa.

Several findings suggest that the mechanism of dosage compensation might be initiated in the ZW female. The MHM RNA is expressed only in females, and is spatially associated with restricted female-specific acetylation of histone H4 [22,23]. Moreover, MHM RNA expression is found in ZW diploid and ZZW triploid chickens, but not in ZZ diploid and ZZZ triploid chickens, which led Teranishi *et al*. [22] to suggest that MHM expression requires the presence of the W chromosome. However, in those studies the presence of the W chromosome was confounded with the Z:A ratio (Z:A ratio of 1 in birds without a W chromosome, and less than 1 for those with a W chromosome), so that it is unclear whether the MHM RNA is activated by the W chromosome in females or inactivated (for example, hypermethylated) based on Z:A ratio in males. Here we find that the M:F ratio of Z genes is correlated better with male expression levels than with female expression levels. Assuming that dosage compensation is equally likely for Z genes with different levels of expression (model I), this pattern suggests that males possess a compensation mechanism. If males reduce gene expression to match that of the female for some Z genes, however, the male Z:A ratio might be expected to be below 1, which is not observed [11]. Thus, model I appears unlikely. Model I would fit a scenario, however, in which the expression of Z genes is increased in both sexes, relative to autosomal genes. An increase in Z gene expression could result from selection pressure in the ZW female in response to gene loss from the W chromosome and differentiation from the Z. Such an effect would have increased the Z:A ratio in males to above 1, and reduced the disparity in expression of Z and A genes in females. At the same time, however, a male-specific reduction in gene expression, restricted to a subset of Z genes, could then have reduced the overall male Z:A ratio to near 1, as we observe in the chicken.

The alternative model II also accounts for the observed pattern of results, if genes selected for dosage compensation have lower average levels of expression than those that are not compensated. That selection makes the male expression level correlate with M:F ratio, and a female-specific compensatory mechanism increases expression of the compensated genes in females to the level of males. The increase in expression of the genes with lower expression levels has the effect of abolishing a positive correlation of M:F ratio with expression level in females (Figure 5). The present data, therefore, are compatible with previous studies suggesting that dosage compensation occurs in females, but is applied more frequently to genes with lower expression values. A male-specific mechanism of dosage compensation is not ruled out, however, and further work is needed to resolve the mechanisms of dosage compensation.

Dosage tolerance and the evolutionary pressure for dosage compensation of Z genes must certainly be related to gene function. Despite the currently incomplete annotation of genes especially in birds, our analysis of Z gene functions is compatible with the idea that compensated and non-compensated genes (or chromosomal regions) are involved in different cellular functions. Genes in the MHM valley were enriched in regulation and developmental functions, whereas genes in the Zq peaks were enriched in catalytic activity and intracellular organelle involvement. Compensated genes on the Z chromosome were enriched in extracellular membrane associated functions like signal transduction and protein binding, compared to intracellular organelle associated functions for non-compensated Z chromosome genes. One interpretation of these findings is that compensated valley and non-valley genes regulate other genes (for example, during development) and, therefore, may be especially sensitive to dosage effects because changes in their dose might propagate through numerous downstream gene networks. In contrast, genes in the peaks and other non-compensated genes participating in intraceulular housekeeping and catalytic activities may be less sensitive to effects of dose. For example, the function of synthetic pathways is not sensitive to the copy number of enzyme genes comprising the pathways [25]. Also, heterozygotes for various traits and metabolic mutations are similar in phenotype to normal homozygotes [25,26]. The presence of many cellular checkpoints in the form of end-product inhibition that control enzyme levels may thus preclude a fine control of such genes at the transcription level [27].

Another factor that might influence the clustering of dosage compensation in the MHM valley is the evolutionary history of this segment. The sex chromosomes of birds and monotremes may have common origins, since the X chromosome of the platypus contains genes in common with the Z chromosome of birds [28,29]. Indeed, homologues of 65% of MHM valley genes and 27% of peak genes are found on the platypus sex chromosome X5, and, therefore, may represent remnants of an ancient precursor to both the monotreme and avian sex chromosomes. The shared evolutionary history of MHM genes on avian and primitive mammalian sex chromosomes raises the question whether genes homologous to the MHM valley are exceptionally well dosage compensated among X genes in monotremes. If so, the evolution of an MHM-specific mechanism of dosage compensation may be ancestral to both birds and monotremes.

How might the MHM valley have accumulated an unusually high concentration of dosage compensated genes? One scenario is that the MHM valley represents the location at which the original sex-determining mutation occurred in birds, producing the proto-W chromosome that was differentiated from the corresponding region of the proto-Z chromosome over a small region [1]. The loss of recombination between the proto-Z and proto-W at this location would have brought the newly hemizygous segment of the Z chromosome under sex-specific selection pressure to compensate Z gene dose. Therefore, the MHM region of the Z chromosome would have been subject to dosage-compensated pressure longer than the rest of the Z chromosome [2,30]. The mammalian X chromosome has similarly been molded by evolutionary forces that produced X strata with different properties depending on time since the divergence of that segment on the X and Y chromosome [30,31]. The oldest stratum shows more complete X-inactivation and contains *SOX3*, the nearest X homologue of the Y-linked testis-determining gene *SRY *[1,30-37]. If the MHM valley represents the oldest part of the Z chromosome, then it might also harbor the site of the original avian W female-determining mutation. It is particularly intriguing, therefore, that the MHM valley includes *DMRT1 *[22], the testis development gene that, if mutated on the W, could have led to *DMRT1 *haploinsufficiency for testis development in ancestral ZW birds [38]. Although this speculation is seductive because it lends weight to the idea that *DMRT1 *is the sex-determining gene, it conflicts with the report that the oldest stratum of chicken Z chromosome is Zq, not Zp where MHM is located [39,40]. One possible resolution of this paradox is that the accurate identification of strata on the chicken Z chromosome may require a comparison of a larger set of Z and W gene sequences than has been available to date, and that the MHM valley is indeed a segment of the oldest stratum that has been translocated to a group of more recently added Zp genes. Alternatively, if the MHM valley is actually a part of a newer segment of the Z, the present results would argue that factors other than time since Z-W divergence may dominate in the evolution of dosage compensation.

## Conclusion

The present results, together with previous studies, show that gene distribution on the Z chromosome is non-random, and that dosage compensated genes occur at higher density in the MHM valley. We propose that compensated genes are the most sensitive to differences in copy number, and that selective compensation of those genes avoids the serious detrimental effects normally associated with aneuploidy. The selectivity of the dosage compensation mechanism may also be reflected in the finding that non-compensated genes have higher average levels of expression than compensated genes in males. The current results are compatible with a female-specific mechanism of dosage compensation of selected genes.

## Materials and methods

### Animals, tissues and microarrays

The preparation of RNA from chicken tissues and microarray data analysis have been described previously [11]. Briefly, brain, heart, and liver tissues were harvested from 20 male and 20 female white leghorn chicken embryos at 14 days of incubation. RNA samples were combined into three to four animals per pool, five pools per sex, and hybridized to Affymetrix Chicken Arrays. The microarray contained probes for over 28,000 chicken genes. Data normalization and filtration were performed in DChip software [11]. To decrease gene redundancy, Affymetrix IDs were combined if they mapped to the same EntrezGeneID. The final dataset of probe sets consisted of 9,692 for brain (465 Z, 9,227 autosomal), 8,737 for liver (415 Z, 8,322 autosomal), and 9,119 for heart (444 Z, 8,675 autosomal). Array data are available from Gene Expression Omnibus [41] (accession numbers GSE6843, GSE6844, GSE6856). Gene positions on the Z chromosome were based on release 2.1 of the chicken genome [42].

### Expression data analysis

Statistical analyses were performed in the statistical environment R 2.0.1 using packages from R and Bioconductor projects [43]. The distribution of M:F ratios on the Z chromosome was analyzed using the gtools R-based package, which computed the running average of M:F ratios with a mean length of 30 genes.

To calculate whether the distribution of M:F ratios along the Z chromosome was random, we calculated: C_low_, the number of consecutive positions of the running average curves in Figures 1b-d that stayed below the 40th percentile of Z gene M:F ratios; and C_high_, the number of consecutive positions that stayed above the 60th percentile. For example, in brain the curve remained below the 40th percentile for 30 gene positions in the valley, and above the 60th percentile for 46 positions on Zq. We then shuffled the M:F ratios of Z genes randomly, calculating C_low _and C_high _each time (using Resampling Stats [44]), to determine the probability that the two runs of values occurred on the same shuffled chromosome. In each tissue (Figures 1b-d), the observed runs of values occurred at *p *< 0.0001.

To compare the level of expression of Z genes in each sex to M:F ratios, we calculated Pearson correlation coefficients (r) between male expression values and the ratios, and female expression values and the ratios, for each tissue. To remove a small number of outliers that have a disproportionate effect on these correlations, we eliminated less than 5% of genes from each tissue for which the male or female expression values differed from the mean by more than 2 standard deviations. Outliers were similarly removed for the calculations in Table 1 but not for the calculations of medians in Figure 3 or the data of Figure 4 and Additional data file 6. We used bootstrap methods [44] to resample with replacement male (or female) expression values on Z chromosomes to determine the probability of finding the mean expression values for compensated or non-compensated genes shown in Table 1.

### Analysis of gene functions and orthologs

We used the DAVID 2.0 and IPA (version 3.0; Ingenuity Systems, Mountain View, CA, USA) [45,46] to identify functional enrichment of different groups of Z chromosome genes. DAVID is a web-based application that allows users to query a database of functional annotations and determine gene enrichment in annotation terms based on Fisher's exact test. We used default parameters in the Chart feature for each data set with highest Gene Ontology term for maximal annotation coverage. Pathway analysis was performed using IPA, which identified most significant biological functions and/or diseases in our datasets. IPA is based on a large number of manually collected relationships between genes from the scientific literature. Gene enrichment was calculated by a right tailed Fisher's exact test. Because IPA is based on mammalian genomes, we converted chicken Z genes to orthologous human genes using data from the Ensembl database version 42 [42].

Gene and population lists were compiled in R and uploaded into DAVID and IPA. Gene lists consisted of compensated, non-compensated, valley, peak, or all Z chromosome genes. Compensated genes were defined as genes with an M:F ratio of less than 1.3 in one tissue, and non-compensated genes were defined as genes with an M:F ratio of greater than 1.5 in one tissue. Genes in the MHM valley were included if they were between 2.5E7 bp and 3.5E7 bp (assembly:WASHUC2). Peak genes were included in the analysis if they were between 5.5E7 and 7.5E7 bp.

## Abbreviations

DAVID, Database for Annotation, Visualization and Integrated Discovery; IPA, Ingenuity Pathway Analysis; M:F, male:female; MHM, male-hypermethylated

## Authors' contributions

Esther Melamed planned and performed analyses, and wrote the first draft of the paper. Arthur P Arnold provided advice on the analyses and interpretation, and helped in writing subsequent drafts of the manuscript.

## Additional data files

The following additional data are available with the online version of this paper. Additional data file 1 is a table listing valley and peak genes expressed in brain, heart, or liver tissues. Additional data file 2 is a table listing gene categories enriched among valley and peak genes using DAVID. Additional data file 3 is a table listing gene categories enriched among valley and peak genes using IPA. Additional data file 4 is a table listing Gene categories enriched among compensated and non-compensated genes using DAVID. Additional data file 5 is a table listing gene categories enriched among compensated and non-compensated genes using IPA. Additional data file 6 is a figure relating gene expression levels to M:F ratio as a function of Z or Chr1 position.

## Supplementary Material

Additional data file 1 Valley and peak genes expressed in brain, heart, or liver tissues. Click here for file

Additional data file 2 Gene categories enriched among valley and peak genes using DAVID. Click here for file

Additional data file 3 Gene categories enriched among valley and peak genes using IPA. Click here for file

Additional data file 4 Gene categories enriched among compensated and non-compensated genes using DAVID. Click here for file

Additional data file 5 Gene categories enriched among compensated and non-compensated genes using IPA. Click here for file

Additional data file 6 **(a) **Brain gene expression levels on the Z chromosome. Top: expression level of individual genes is plotted by gene position for males and females. Middle: the running average of 30 M:F ratios is plotted according to median gene position. Bottom: the running average of expression level of 30 genes is plotted relative to median gene position. The MHM valley and Zq peak are not characterized by unusually high or low expression values. A similar conclusion emerges from analyses of gene expression in heart and liver, and from analyses in which a small percentage of genes with very high expression values are removed so as to limit their disproportionate effect on running averages (data not shown). **(b) **Above: the running average of 30 brain M:F ratios from chromosome 1, plotted relative to median gene position. Below: the running average brain expression values for 30 genes in males and females. M:F ratios are quite different for chromosomes 1 and Z, even though the levels of gene expression were similar (for example, in male brain Z gene expression ranged from 14-6,334 (mean 335) versus 11-6,451 (mean 324) for chromosome 1). Click here for file
