# Supplementary figures and images for: Regional differences in dosage compensation on the chicken Z chromosome
Source: Genome Biol. 2007 Sep 27;8(9):R202. doi: 10.1186/gb-2007-8-9-r202 (PMC2375040; doi:10.1186/gb-2007-8-9-r202)

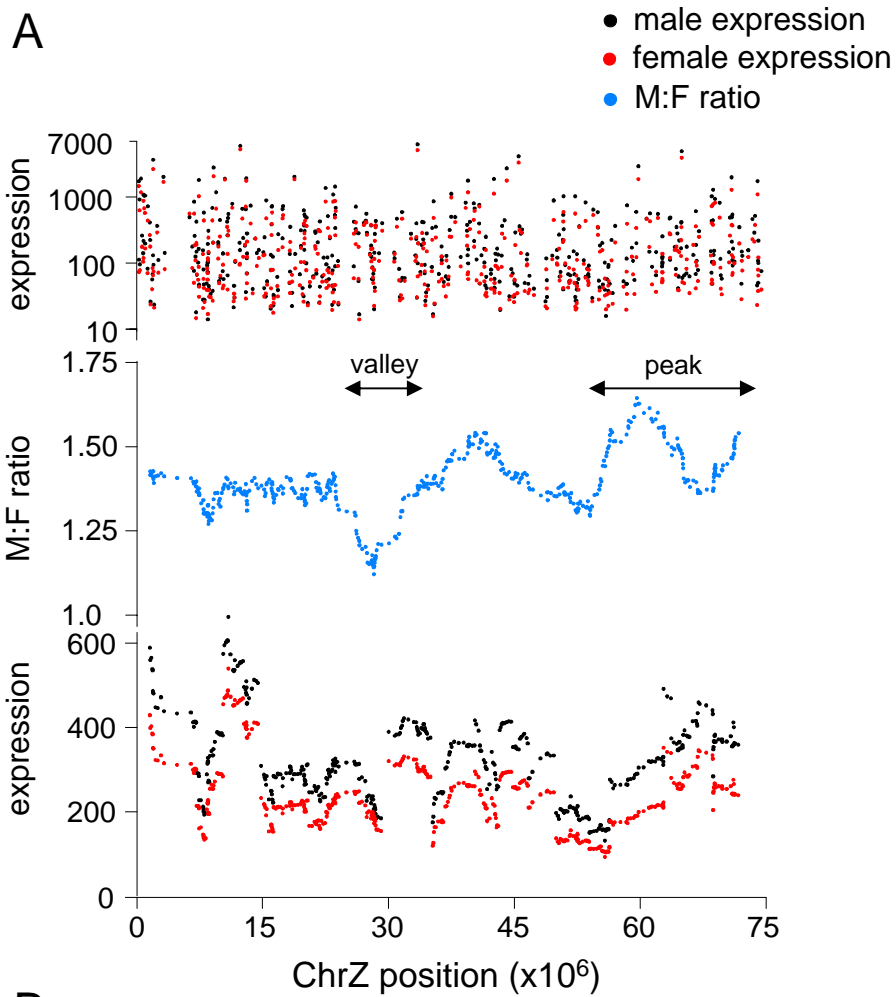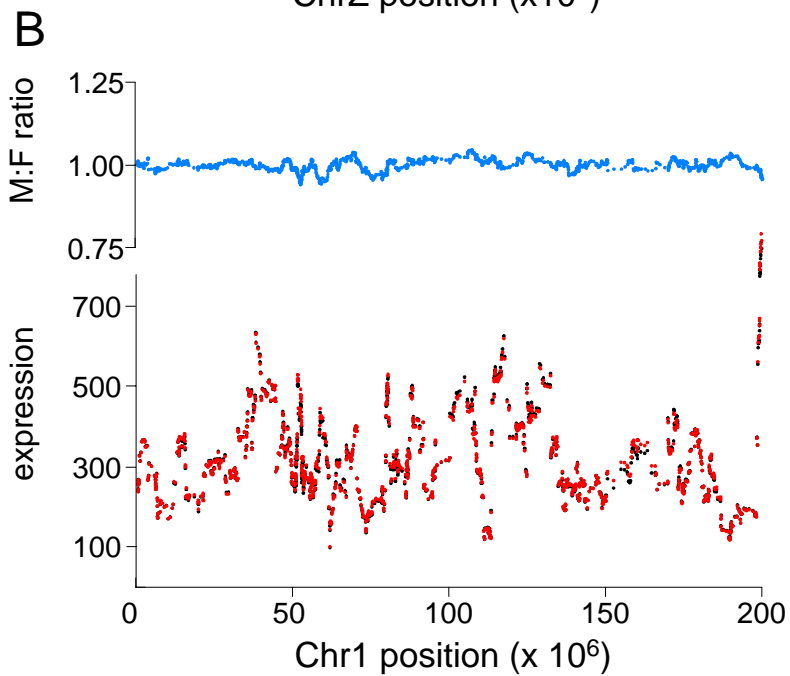

Supplementary  
Figure 1

Supplement: Additional data file 6 — (a) Brain gene expression levels on the Z chromosome. Top: expression level of individual genes is plotted by gene position for males and females. Middle: the running average of 30 M:F ratios is plotted according to median gene position. Bottom: the running average of expression level of 30 genes is plotted relative to median gene position. The MHM valley and Zq peak are not characterized by unusually high or low expression values. A similar conclusion emerges from analyses of gene expression in heart and liver, and from analyses in which a small percentage of genes with very high expression values are removed so as to limit their disproportionate effect on running averages (data not shown). (b) Above: the running average of 30 brain M:F ratios from chromosome 1, plotted relative to median gene position. Below: the running average brain expression values for 30 genes in males and females. M:F ratios are quite different for chromosomes 1 and Z, even though the levels of gene expression were similar (for example, in male brain Z gene expression ranged from 14-6,334 (mean 335) versus 11-6,451 (mean 324) for chromosome 1). [file gb-2007-8-9-r202-S6.pdf]
